# Inhibition of visfatin alleviates sepsis-induced intestinal damage by inhibiting Hippo signaling pathway

**DOI:** 10.1007/s00011-022-01593-z

**Published:** 2022-06-22

**Authors:** Zhong-Shen Kuang, Yu-Xin Leng, Ning Yang, Zheng-Qian Li, Ya-Nan Zong, Deng-Yang Han, Yue Li, Jin-Dan He, Xing-Ning Mi, Zhu-Kai Cong, Xi Zhu, Chang-Yi Wu, Xiang-Yang Guo

**Affiliations:** 1 Department of Anesthesiology, Peking University Third Hospital, No. 49, North Garden Street, Haidian District, Beijing, 100191 China; 2 Department of Critical Care Medicine, Peking University Third Hospital, Beijing, 100191 China

**Keywords:** Visfatin, Sepsis, FK866, Intestine, Inflammation, Hippo

## Abstract

**Background:**

The aim of this study is to investigate role of Visfatin, one of the pro-inflammatory adipokines, in sepsis-induced intestinal injury and to clarify the potential mechanism.

**Methods:**

C57BL/6 mice underwent cecal ligation and puncture (CLP) surgery to establish sepsis model in vivo. Intestinal epithelial cells were stimulated with LPS to mimic sepsis-induced intestinal injury in vitro. FK866 (the inhibitor of Visfatin) with or without XMU-MP-1 (the inhibitor of Hippo signaling) was applied for treatment. The expression levels of Visfatin, NF-κB and Hippo signaling pathways-related proteins were detected by western blot or immunohistochemistry. The intestinal cell apoptosis and intestinal injury were investigated by TUNEL staining and H&E staining, respectively. ELISA was used to determine the production of inflammatory cytokines.

**Results:**

The expression of Visfatin increased in CLP mice. FK866 reduced intestinal pathological injury, inflammatory cytokines production, and intestinal cell apoptosis in sepsis mice. Meanwhile, FK866 affected NF-κB and Hippo signaling pathways. Additionally, the effects of FK866 on inflammatory response, apoptosis, Hippo signaling and NF-κB signaling were partly abolished by XMU-MP-1, the inhibitor of Hippo signaling. In vitro experiments also revealed that FK866 exhibited a protective role against LPS-induced inflammatory response and apoptosis in intestinal cells, as well as regulating NF-κB and Hippo signaling, whereas addition of XMU-MP-1 weakened the protective effects of FK866.

**Conclusion:**

In short, this study demonstrated that inhibition of Visfatin might alleviate sepsis-induced intestinal injury through Hippo signaling pathway, supporting a further research on Visfatin as a therapeutic target.

## Introduction

Sepsis is a systemic inflammatory response syndrome mainly induced by major surgery, severe burns or trauma infection [1]. The disease is characterized by high incidence, rapid progression, difficult treatment and high fatality rate [2]. The gut which is the largest repository of bacteria in the body has been hypothesized to the motor of sepsis [3]. The intestinal barrier, consisting of intestinal epithelial cells, is the body’s largest immune organ that prevents bacteria and endotoxin from entering the bloodstream from the gut. Emerging information suggests that sepsis leads to a variety of disorders of intestinal epithelial cells, such as cytokines overproduction, epithelial apoptosis and barrier dysfunction [4]. Prevention of intestinal apoptosis has been demonstrated to improve the survival rate of septic mice [5]. Therefore, an in-depth study of the underlying pathogenesis of intestinal injury in sepsis is of great significance for the treatment and prognosis of this disease.

Visfatin, also known as pre-B-cell colony-enhancing factor (PBEF) and nicotinamide phosphatidyl transferase (Nampt), is a 52 kDa molecule that was first identified as a cytokine involved in B-cell maturation and is highly expressed in bone marrow, liver, and muscle[6, 7]. Visfatin is secreted by a variety of cell types, including lymphocytes, neutrophils, macrophages, and epithelial cells [8], and is closely associated with various types of inflammatory diseases, including psoriasis [9], atherosclerosis [10] and acute lung injury [11], as well as sepsis [12]. Recently, a high level of serum Visfatin was observed in patients with sepsis, which was also positively associated with a high mortality, indicating that Visfatin might serve as a circulating biomarker of sepsis disease [13]. Nevertheless, studies on the specific role of Visfatin in sepsis and its mechanism have not been reported so far, nor has there been any study on its relationship with intestinal damage caused by sepsis.

A wide spectrum of literature suggest that Visfatin can be considered as a novel inflammatory cytokine as it promotes the production of pro-inflammatory cytokines such as IL-6, IL-1β and TNF-α [14]. The activation of the NF-κB signaling pathway, which is critically involved in the pathogenesis of sepsis, plays an important role during the production and secretion of pro-inflammatory factors induced by Visfatin [15]. The classic Hippo pathway, highly evolutionarily conserved in mammals, is a crucial regulator to control organ size and tissue homeostasis, contributing to a coordinated balance between cell proliferation, differentiation and apoptosis [16]. Yes-associated protein (YAP) is a key downstream effector of Hippo signaling pathway, which has been found to play a critical role in inflammatory disorders, such as lung inflammation, liver inflammatory injury, and intestinal inflammation [17–19]. In addition, the activation of Hippo signaling pathway and the elevated expression of YAP are reported to be associated with the progression of sepsis, and Hippo/YAP pathway was essential for sepsis-induced inflammation and organ failure [20, 21]. To the best of our knowledge, the direct association between Visfatin and Hippo/YAP pathway in sepsis-related intestinal injury has not been reported yet; however, Visfatin was evidenced to serve a role linked to Sirt1/YAP pathway in obesity-associated gastric cancer, which partly indicates the connection between Visfatin and Hippo/YAP signaling [22]. In view of the evidence above, whether Hippo/YAP signaling is involved in the specific role of Visfatin in sepsis-induced intestinal injury raises our interests, which is deserved to be explored.

In the present study, a sepsis mouse model was established by cecal ligation and puncture and a cell model was established by lipopolysaccharide administration to intestinal epithelial cells, and the role of Visfatin in sepsis-caused intestinal injury was assessed. We aimed to investigate the role and the underlying mechanism of Visfatin in sepsis-caused intestinal injury, thus providing an important theoretical basis for the clinical treatment of intestinal injury caused by sepsis.

## Materials and methods

### Animals

Male C57BL/six mice aged 8–10 weeks were obtained from Peking University Health Science Center and housed in a temperature and light controlled room. All animals were fed ad libitum. All animal protocols conformed to the guide for the care and use of laboratory animals and were approved by the institutional animal care and use committee of Peking university third hospital. For generation of sepsis mode in vivo, mice underwent cecal ligation and puncture (CLP) surgery. To examine the role of Visfatin in intestinal injury caused by sepsis, mice were intraperitoneally injected by FK866 (the inhibitor of Visfatin; 20 mg/kg; Cayman Chemical, Michigan, USA) with or without XMU-MP-1 (the inhibitor of Hippo signaling; 3 mg/kg; MedChem Express) 3 h prior to surgery.

### The model of cecal ligation and puncture (CLP)

Sepsis was induced following some modifications of previously published studies [23, 24]. Mice were anesthetized with 2% isoflurane (Sigma-Aldrich) inhalation. A 1 cm incision was made to the abdomen, and the cecum was exteriorized and ligated 2 cm from the tip with 3-0 silk suture. Then, a 20-gauge needle was used to make a single puncture to the distal cecum and a small amount of fecal contents was extruded. The cecum was replaced into the abdominal cavity and the exposed abdominal wall was closed with running 4-0 silk suture. Only laparotomy was performed in the mice of sham-operated group, and their cecum was not ligated or punctured. Finally, animals were resuscitated with 1 ml saline subcutaneously. 24 h and 48 h following the CLP and sham-procedure, a clinical disease score was applied to evaluate the degree of CLP-induced intestinal injury as described previously [25].

### Enzyme-linked immunosorbent assay (ELISA)

The content of TNF-α, IL-1β, IL-6 and IL-17A in serum was assessed by their corresponding ELISA kits (R&D Systems, Minneapolis, MN, USA) according to the manufacturer’s instructions.

### H&E and immunohistochemistry

Small intestine tissue was fixed with 4% paraformaldehyde overnight at room temperature, embedded in paraffin, sliced into 5 μm sections and deparaffinized. The sections were stained by hematoxylin and eosin staining (H&E). The degree of injury was evaluated using the criteria of Chiu’s score method [26]. For immunohistochemistry, the sections were incubated with a primary antibody against Visfatin (1:100; ab236874, Abcam, Cambridge, MA, USA) overnight at 4 °C and then with second antibody at room temperature. Stained tissues were examined under a light microscope (BX53, Olympus, Japan).

### TUNEL assay

The TUNEL assay was performed to detect intestinal tissue apoptosis according to the manufacturer’s instructions. The paraffin-embedded sections were permeabilized with xylene at 4 °C for 2 min and incubated with TUNEL reagent for 1 h at 37 °C. Photomicrographs were obtained using a fluorescence microscope (Eclipse E800; Nikon, Tokyo, Japan) [24].

### Western blot

Proteins were extracted from intestinal tissues by RIPA lysis buffer (Beyotime Biotechnology, Shanghai, China) containing protease inhibitors, phosphatase inhibitors and PMSF. The concentrations of the samples were determined by BCA assay (KeyGEN Biotech. Co. Ltd, Nanjing, China). Total proteins were separated from the samples by 8–12% SDS-PAGE and transferred onto polyvinylidene fluoride membranes. After that, the membranes were blocked with 5% (w/v) nonfat dry milk in Tris-buffered saline for 2 h at room temperature and then incubated overnight with primary antibodies overnight at 4 ℃. Subsequently, the membranes were incubated with secondary antibodies at room temperature for 1.5 h, and specific bands were detected by Bio-Rad ECL system (Hercules, CA, USA).

### RNA extraction and real-time quantitative PCR (RT-qPCR)

Total RNA was extracted from intestinal tissues by TRIzol reagent (Invitrogen, Carlsbad, CA, USA). Subsequently, the total RNA was reverse transcribed into cDNA using the PrimeScript RT Master Mix kit (Takara, Japan). RT-qPCR was then performed using the SYBR green mix (Applied Biosystems, USA) on a 7500 fast real-time PCR system (Applied Biosystems, USA) in line with the manufacturer’s protocol. GAPDH was used as an internal reference gene for normalization. Relative mRNA level was calculated using the 2^−△△*C*t^ method.

### Extraction and culture of intestinal epithelial cells

The fetus was taken from ICR pregnant mice under aseptic conditions. And the small intestine of the fetus was cut into pieces smaller than 1 mm^3^. Serum-free DMEM/F12 was used to wash small intestinal tissue fragments until the supernatant was clear. Tissues were suspended in DMEM/F12 medium containing 5% FBS, and areas containing fibroblasts were scraped off. The epithelial cells were then digested with trypsin, resuspended and cultured in DMEM/F12 medium containing penicillin (100 U/mL), streptomycin (100 μg/mL), Gln (2 mmol/L), insulin (5 μg/ml) and EGF (10 ng/ml). Afterwards, cells stimulated with lipopolysaccharide (LPS; 1 µg/ml) for 24 h to simulate sepsis-induced intestinal injury in vitro [27, 28]. FK866 (1 μM) and/or XMU-MP-1 (1 μM) were used for treatment.

### Statistical analysis

The results are presented as the mean ± SD. Differences among groups were calculated by one-way analysis of variance (ANOVA) followed by Tukey’s post hoc test. All data were analyzed using Prism 8.0 (GraphPad Software, USA). Statistical significance was determined as *p* < 0.05.

## Results

### Visfatin expression is elevated in the small intestine of septic mice

**Fig. 1:**
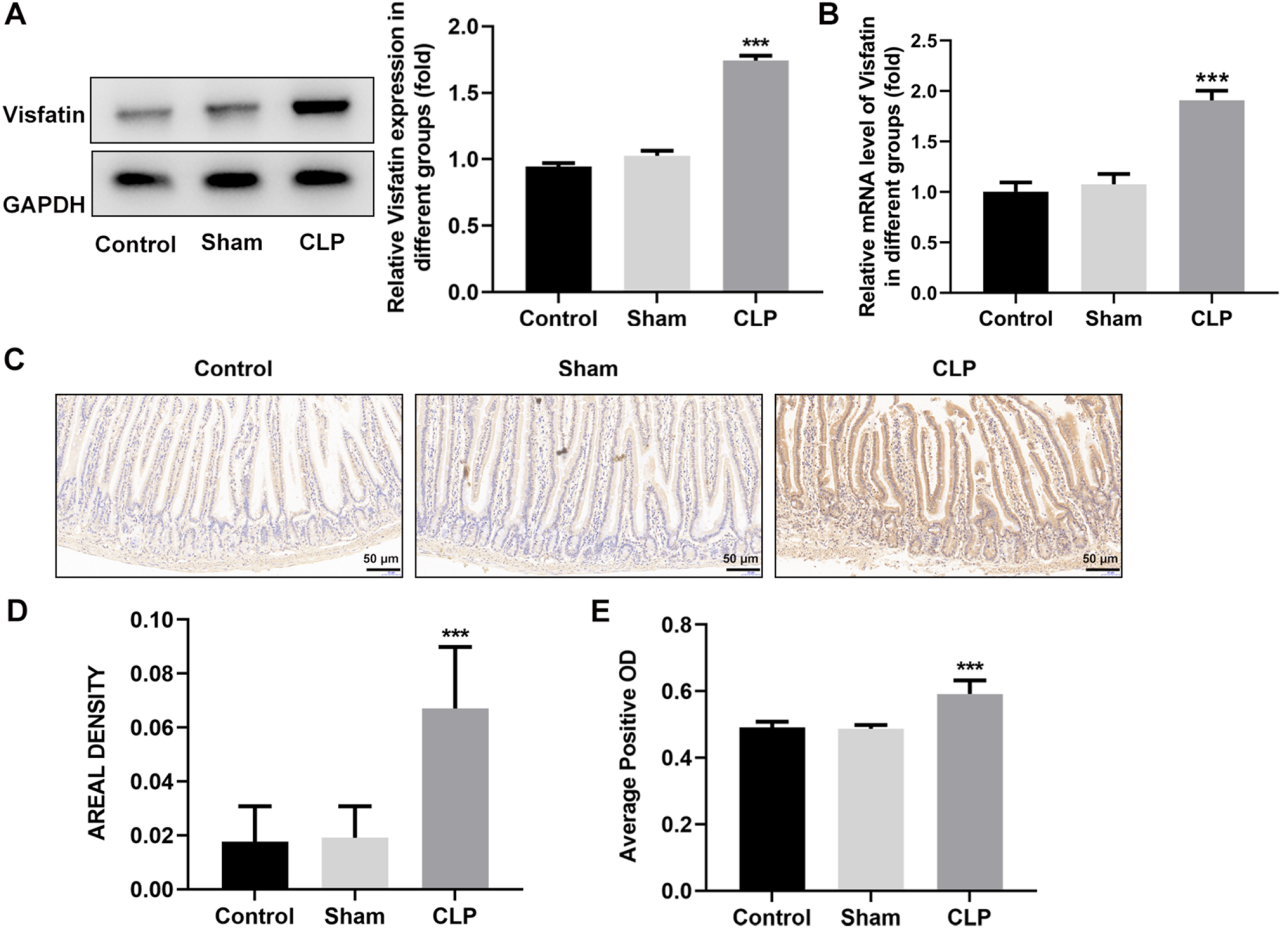
Visfatin expression is elevated in the small intestine of septic mice. **A** C57BL/6 mice underwent cecal ligation and puncture (CLP) surgery to establish sepsis model in vivo. The protein expression of Visfatin was measured by western blot. **B** The mRNA level of Visfatin was measured by RT-qPCR. **C** Immunohistochemistry staining of Visfatin in the small intestine. **D**, **E** The areal density and average optical density was calculated according to Immunohistochemistry staining. ***p < 0.001 vs Sham

Visfatin-1 expression is of high abundance in intestinal tract and epithelial cells. Here, results from western blot and RT-qPCR showed that the expression of Visfatin of intestinal epithelia in the Control group and the Sham group was similar, and and both of the protein expression and mRNA level of Visfatin in the CLP group was significantly increased compared to the Sham group (Fig. 1A, B). Immunohistochemical staining showed that the obvious brown intestinal epithelia in CLP indicated a high expression of Visfatin, compared to that in the sham group (Fig. 1C). In addition, the areal density and average optical density of the CLP group were much higher than those of the Control group and the Sham group, indicating a marked increase of Visfatin expression in the sepsis mouse model (Fig. 1D, E).

### FK866 inhibits Visfatin expression and reduces sepsis scores in septic mice

**Fig. 2:**
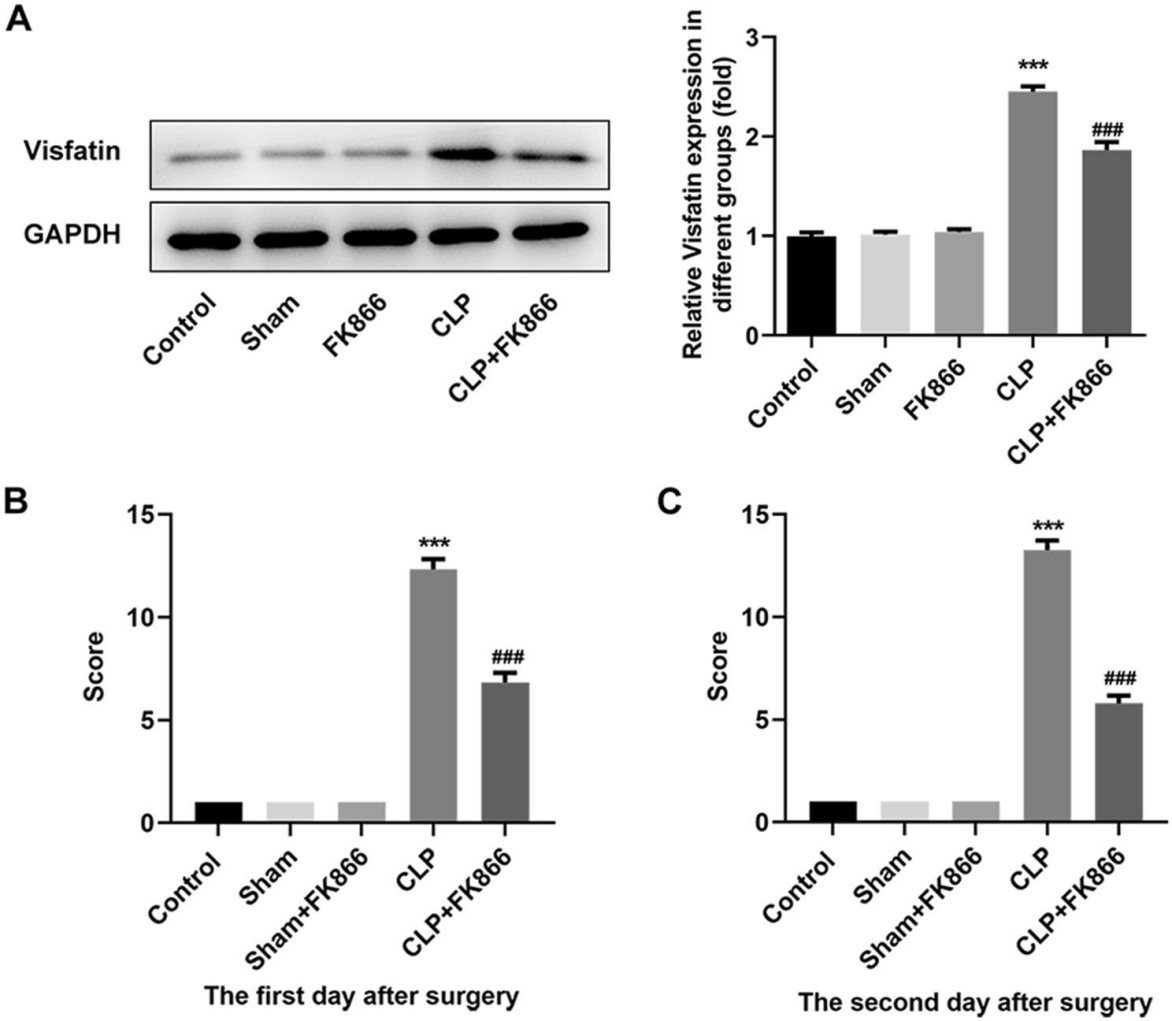
Visfatin inhibitor FK866 can reduce disease scores and mortality in septic mice. **A** The septic mice were treated with or without FK866, the inhibitor of Visfatin. The protein expression of Visfatin in each group was detected using western blot. **B**, **C** The disease scores of mice in different groups on the first and second days. ***p < 0.001 vs Sham; ^###^*p* < 0.001 vs CLP

Next, to explore the effect of Visfatin in sepsis-induced intestinal injury, the septic mice were administrated with FK866, the inhibitor of Visfatin. As shown in Fig. 2A, the administration with FK866 did not affect the expression of Visfatin in sham mice, but greatly reduced the expression of Visfatin in septic mice. Subsequently, as shown in Fig. 2B, C, the disease scores of mice in the CLP model group on the first and second days after surgery were significantly increased compared with the Sham group. The disease score of the mice in the FK866 intervention group decreased compared with CLP group. It shows that inhibiting Visfatin can relieve the symptoms of sepsis mice.

### FK866 alleviates intestinal pathological injury, inflammation and cell apoptosis in septic mice

**Fig. 3:**
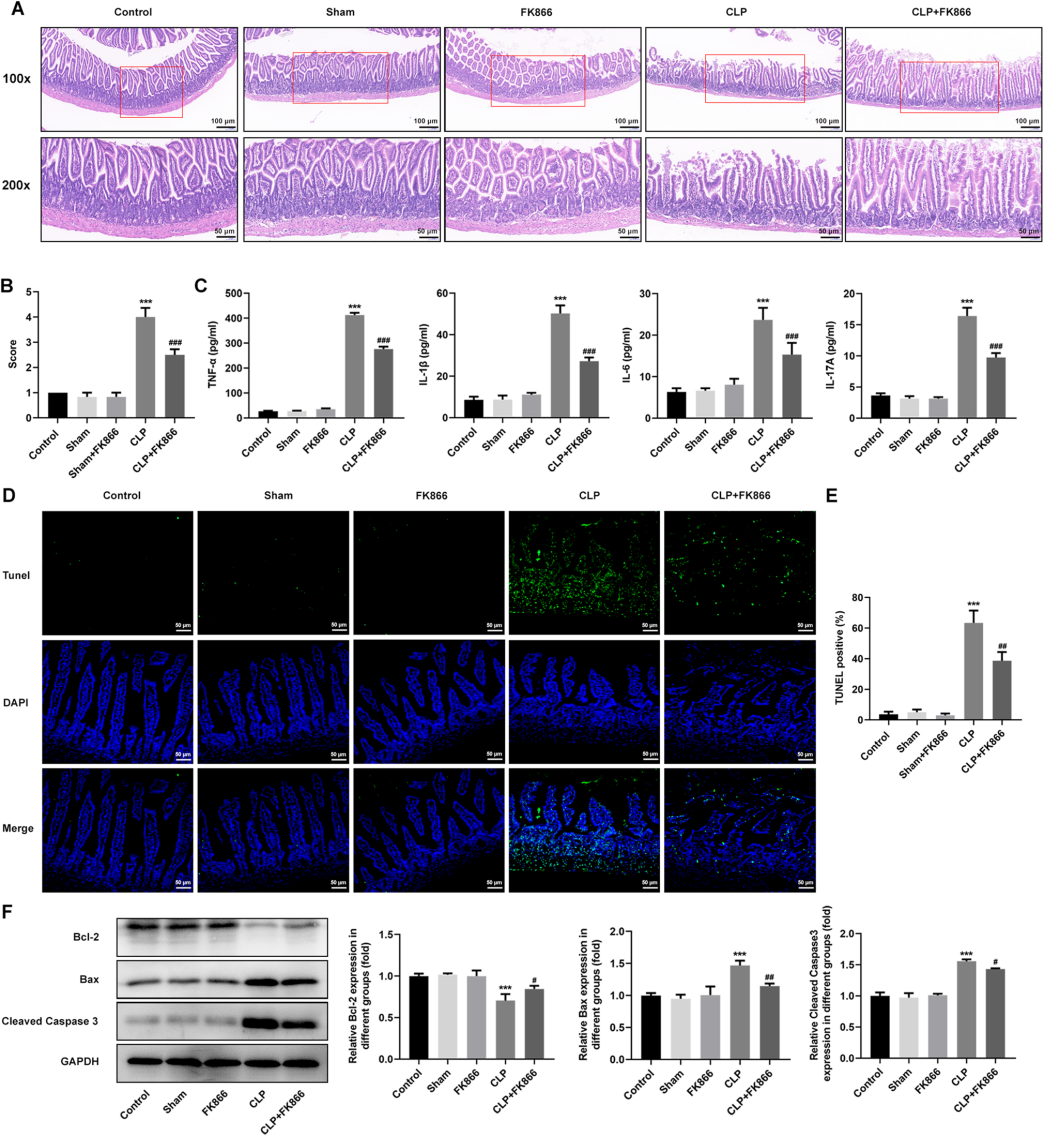
FK866 alleviates intestinal pathological injury, inflammation and cell apoptosis in septic mice. **A**, **B** The pathological injury of intestine was detected using H&E staining. **C** The concentration of serum TNF-α, IL-6, IL-1β and IL-17A was determined using their ELISA kit. **D**, **E** Cell apoptosis of intestine was assessed by TUNEL staining. Nuclei were stained with DAPI. **F** The apoptosis-related proteins were measured using western blot. ***p < 0.001 vs Sham; ^#^*p* < 0.05, ^##^*p* < 0.01, and ^###^*p* < 0.001 vs CLP

The H&E results showed that the control group has relatively complete structure of intestinal tissue, but extensive pathological changes occurred in CLP group, including the irregular arrangement and shrinkage of the intestinal villi, and narrowed thickness of basement membrane, indicating a serious intestinal damage in sepsis mice. Notably, the injury score indirectly reflected severe injury in CLP group and an improvement upon FK866 treatment, suggesting that FK866 treatment partly alleviated intestinal pathological injury in sepsis mice (Fig. 3A, B). Then, the detection of serum inflammatory factor levels showed that the expression of TNF-α, IL-1β, IL-6 and IL-17A in the CLP group was obviously elevated compared with the Control group, and that the expression level of TNF-α, IL-1β, IL-6 and IL-17A was largely reduced after administration of FK866 (Fig. 3C). In addition, Tunel results showed that compared with the sham group, the small intestinal epithelial cells of the sepsis model exhibited obvious apoptosis, and that inhibition of Visfatin could reduce cell apoptosis (Fig. 3D, E). Moreover, FK866 treatment obviously abolished the downregulated expression of Bcl-2 and the upregulated expression of Bax and Cleaved caspase-3 in septic mice (Fig. 3F), further demonstrating the anti-apoptotic activity of FK866 in intestinal tissue of septic mice.

### FK866 affects Hippo/NF-κB signaling pathway in septic mice

**Fig. 4:**
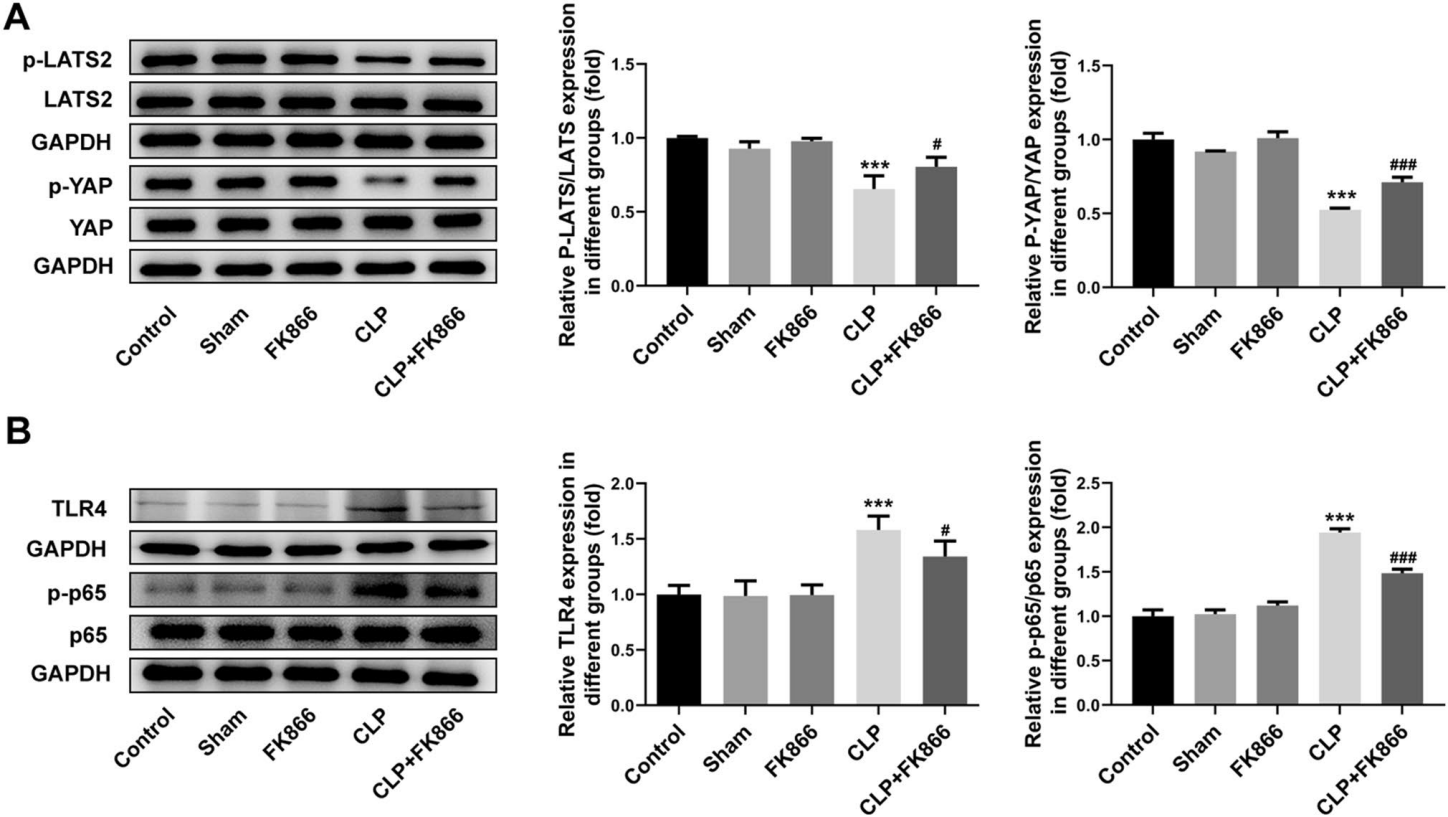
FK866 affects Hippo and NF-κB signaling pathways in septic mice. **A** The protein expression of p-LATS2, LATS2, p-YAP and YAP was determined by western blot. **B** The protein expression of p-p65, p65 and TLR4 was measured using western blot. ***p < 0.001 vs Sham; ^#^*p* < 0.05, ^###^*p* < 0.001 vs CLP

As shown in Fig. 4A, compared with the Sham group, the expression of YAP and LATS2 in the CLP model group did not change, whereas the phosphorylation levels of YAP (p-YAP) and p-LATS2 significantly decreased; however, the expression changes of p-YAP and p-LATS2 were partly abolished by FK866 intervention. What’s more, the expression of p-p65 in intestinal tissues increased significantly in the CLP group and decreased significantly after FK866 intervention. The expression of TLR4 in the CLP model group also increased significantly, which was then partly abolished by FK866 treatment (Fig. 4B).

### XMU-MP-1 treatment weakens the protective role of FK866 in septic mice

**Fig. 5:**
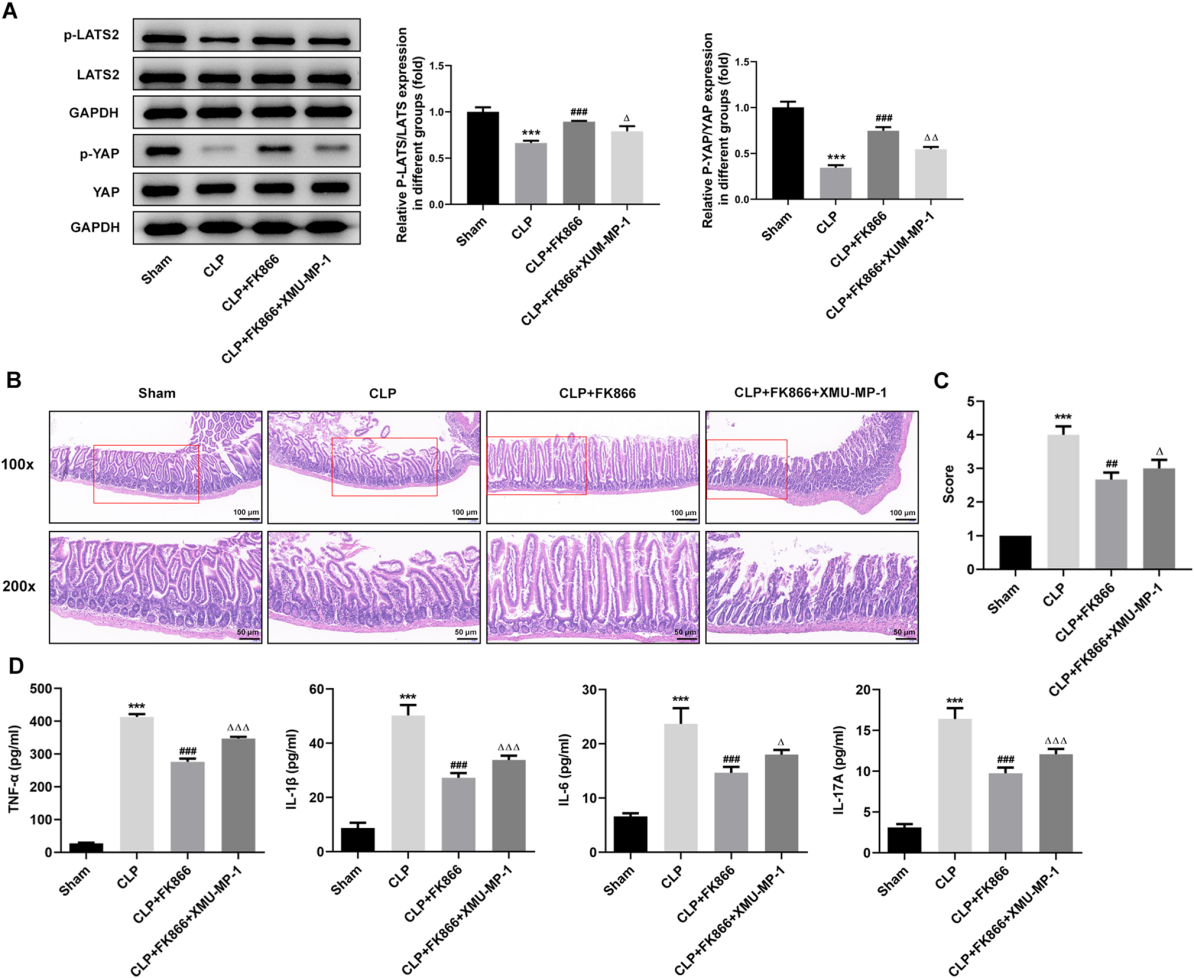
XMU-MP-1 treatment weakens the protective role of FK866 in septic mice. **A** The septic mice were treated with FK886 with or without XMU-MP-1, the inhibitor of Hippo signaling. Then, the protein expression of p-LATS2, LATS2, p-YAP and YAP was determined by western blot. **B**, **C** The pathological injury of intestine detected using H&E staining. **D** The concentration of serum TNF-α, IL-6, IL-1β and IL-17A was determined using their ELISA kit. ***p < 0.001 vs Sham; ^###^*p* < 0.001 vs CLP; ^△^*p* < 0.05, ^△△^*p* < 0.01, and ^△△△^*p* < 0.001 vs CLP + FK866

As a crucial role of Hippo signaling involved in intestinal injury in FK866-treated septic mice as aforementioned, the FK866-treated septic mice were additionally treated with XMU-MP-1, the inhibitor of Hippo pathway, to further explore whether Hippo signaling is an indispensable pathway by which FK866 exerted its protective effects on intestinal injury in septic mice. Firstly, as exhibited in Fig. 5A, compared to FK866-treated septic mice, additional treatment of XMU-MP-1 greatly reduced the p-LATS2/t-LATS2 and p-YAP/t-YAP, indicating that inhibition of Hippo signaling induced YAP activity upon XMU-MP-1 treatment. Afterwards, a series of indexes concerning on intestinal injury were detected to determine the importance of Hippo signaling involved in the protection of FK866 in septic mice. As shown in Fig. 5B–D, the protective effects of FK866 on alleviating intestinal damage and inflammatory response were greatly weakened by XMU-MP-1 treatment, suggesting that FK866 might exert its effects on attenuating sepsis-induced intestinal injury by activating Hippo signaling pathway.

### FK866 attenuates inflammatory response and cell apoptosis in LPS-induced intestinal epithelial cells through Hippo signaling pathway

**Fig. 6:**
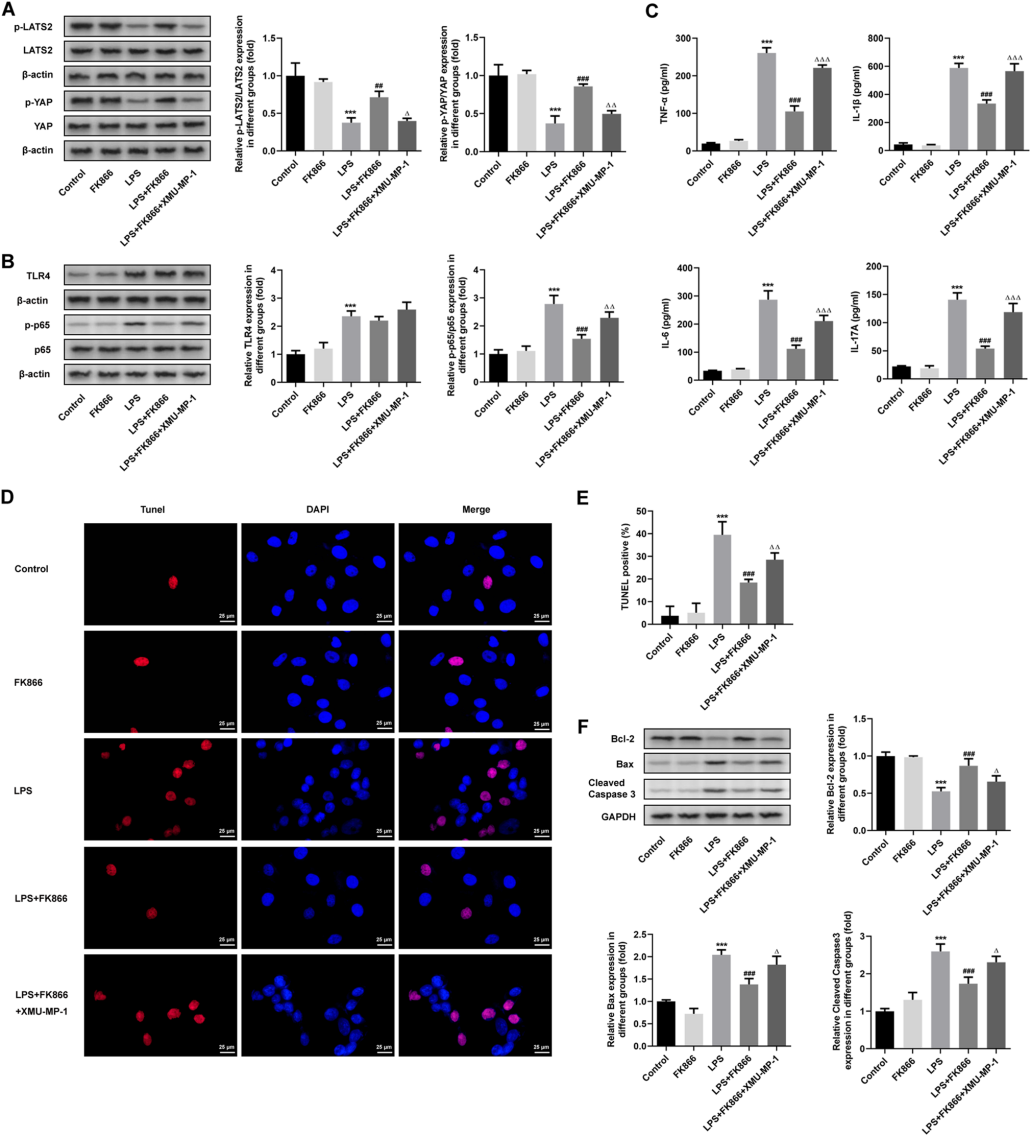
FK866 attenuates inflammatory response and cell apoptosis in LPS-induced intestinal epithelial cells through Hippo signaling pathway. **A** LPS-induced intestinal epithelial cells were used to mimic sepsis-induced intestinal injury model in vitro. The protein expression of p-YAP, YAP, p-LATS2 and LATS2 in intestinal epithelial cells was measured by western blot. **B** The expression NF-κB-related proteins in intestinal epithelial cells was measured by western blot. **C** The concentration of TNF-α, IL-6, IL-1β and IL-17A was determined using their ELISA kit. **D**, **E** Cell apoptosis of intestine was assessed by TUNEL staining. **F** The apoptosis-related proteins were measured using western blot. ***p < 0.001 vs Control; ^##^*p* < 0.01, ^###^*p* < 0.001 vs LPS; ^△^*p* < 0.05, ^△△^*p* < 0.01, and ^△△△^*p* < 0.001 vs LPS + FK866

Finally, to further verify the findings above, the primary intestinal epithelial cells from mice were cultured, followed by stimulation with LPS to mimic sepsis-induced intestinal injury model in vitro. Compared with the Control group, the p-YAP/t-YAP and p-LATS2/t-LATS2 in the LPS group were significantly decreased, while FK866 significantly increased p-YAP/t-YAP and p-LATS2/t-LATS2, which was then weakened by XMU-MP-1 treatment (Fig. 6A). In addition, LPS stimulation significantly increased the protein expression of TLR4 and p-p65. After administration of FK866, the protein expression of p-p65 was significantly reduced compared to LPS group, which was partly hindered by the treatment of XMU-MP-1. However, the protein expressions of total p65 and TLR4 did not change upon FK866 or XMU-MP-1 treatment (Fig. 6B).

Subsequently, the inflammatory cytokines and the apoptosis level in intestinal epithelial cells were detected. As shown in Fig. 6C, the pro-inflammatory cytokines, including TNF-α, IL-6, IL-1β and IL-17A, were notably elevated upon LPS stimulation in intestinal epithelial cells. FK866 treatment significantly reduced the production of these inflammatory cytokines, which was partly abolished by XMU-MP-1 treatment. In addition, apoptotic cells were increased upon LPS stimulation, as well as the downregulated protein expression of Bcl-2 and upregulated protein expression of Bax and Cleaved caspase-3, demonstrating a huge increase of apoptosis in LPS-stimulated intestinal epithelial cells. However, this increase was weakened by FK866 treatment, which was hindered by co-treatment with FK866 and XMU-MP-1 (Fig. 6D–F). Taken together, these results suggested that FK866 could alleviate intestinal injury by inhibiting LPS-induced inflammatory response and cell apoptosis through Hippo signaling pathway in vitro.

## Discussion

Sepsis is a life-threatening syndrome with a high mortality worldwide. The intestine has always been regarded to play a critical role in the pathophysiology of sepsis and is usually considered as the “motor” of this systemic inflammatory response syndrome [29]. Visfatin, an adipocytokine with metabolic and immune functions, has been evidenced to directly participate into the pathogenesis of critical illness and systemic inflammation [13]. To our knowledge, there have been no studies focusing on the effect of Visfatin on sepsis-related diseases. This was the first study which investigated (a) the role of Visfatin in sepsis-induced intestinal injury, (b) the effect of Visfatin on sepsis-induced intestinal pathological injury, as well as the intestinal cell apoptosis and inflammation, and (c) the potential signaling pathways that were involved in the functions of Visfatin. Our results clearly revealed that Visfatin was highly expressed in intestinal tissues of septic mice. Then, FK866, the inhibitor of Visfatin, was used for the treatment of sepsis-induced intestinal injury model in vivo and in vitro in subsequent experiments. A series of data demonstrated that FK866 not only alleviated the pathological symptoms in septic mice, but also inhibited inflammatory cytokines production and intestinal cell apoptosis. In addition, FK866 inhibited NF-κB while activated Hippo signaling pathway, and blockage of Hippo pathway weakened the protective role of FK866 in septic mice. Thus, these results suggested that Visfatin might be a potential target for attenuating sepsis-induced intestinal injury, and provided a mechanistic insight into the regulatory role of Visfatin in sepsis.

Sepsis leads to multiple derangements in the intestinal epithelium, such as epithelial apoptosis and cytokines release [4]. The increasing evidence showed that targeting inflammation and apoptosis might be effective strategies for the treatment of sepsis-induced organ disorders. For example, Wang et al. investigated the effect of simvastatin on sepsis-induced intestinal injury, and it was found that simvastatin could ameliorate the intestinal injury caused by sepsis partly by reducing the levels of inflammatory cytokines [30]; Guo et al. showed that Esmolol played a protective role in the early stage sepsis rats by inhibiting inflammation and apoptosis in the intestinal tissue [31]. In the present study, the apoptotic cells in the intestine of septic mice and the intestinal epithelial cells stimulated by LPS were markedly increased, which were partly abolished by the administration of FK866. Furthermore, our study demonstrated that FK866 significantly reduced the concentrations of TNF-α, IL-6, IL-1β and IL-17A of septic mice or LPS-induced intestinal epithelial cells, revealing the anti-inflammatory property of FK866. Thus, FK866 might protect septic mice against intestinal injury via inhibiting inflammation and intestinal cell apoptosis.

It is known that NF-κB is critically involved in the pathogenesis of sepsis. In septic patients, the activity of NF-κB was markedly increased, and the greater levels of NF-κB were associated with a higher rate of mortality and worse clinical outcome [32]. Moreover, activation of NF-κB is involved in or contributes to the development of sepsis-induced intestinal barrier dysfunction [33]. However, whether NF-κB activation participates into the biological function of Visfatin in sepsis-induced intestinal injury remains unclear. Studies have shown that NF-κB activation plays an important role in immune response [34]. NF-κB activation is the consequence of NF-κB inhibitor (IκB) phosphorylation and degradation, leading to the translocation of NF-κB into the nucleus and activation of the downstream inflammatory cascade reaction [35]. TLR4 acts as an essential LPS signaling receptor, and TLR4/NF-κB signaling pathway has been reported to be involved in the pathology of septic experimental animal’s inflammatory injury [36]. Interestingly, we found a significant upregulation of TLR4 both in septic mice and LPS-induced intestinal cells, as well as the phosphorylation of NF-κB p65. In addition, FK866 exhibited an obvious inhibition on the phosphorylation of NF-κB p65 and IκBα, but not affected the expression of TLR4 greatly, indicating that FK866 suppressed the activation of NF-κB p65, which might partly account for the inhibition of inflammation.

The core of the Hippo signaling pathway includes STE20 family protein kinases (MST1/2) and large tumor suppressor kinase (LATS1/2). YAP is a key downstream effector of Hippo signaling pathway. Upon activation of the Hippo pathway, MST kinases will phosphorylate and activate LATS1/2, which in turn phosphorylates YAP, causing it to be retained in the cytoplasm for degradation. Once the Hippo pathway is turned off, which leaves YAP un-phosphorylated, YAP can translocate into the nuclear and bind to transcription factors, thus activating the expression of target genes important for various cellular processes [16, 20, 37–39]. Meanwhile YAP is necessary for NF-κB activation, as YAP can interact with NF-κB subunit p65 promoter to enhance NF-κB signaling, thus regulating inflammatory response [40, 41]. In addition, the LPS-induced activation of NF-κB was enhanced in MST1/2 deficient bone marrow-derived macrophages [42]. These findings reveal a crucial Hippo/YAP/NF-κB signaling pathway crosstalk in inflammation of the body. In our research, we measured the effects of Visfatin on the Hippo/YAP and NF-κB signaling pathways. It was found that the levels of p-LATS2 and p-YAP were downregulated in septic mice and LPS-induced intestinal cells, indicating an inactivation of Hippo signaling pathway in sepsis, whereas FK886 had the ability to partly restore the activity of Hippo signaling, suggesting that FK886 might stimulate the activation of Hippo signaling, followed by YAP phosphorylation and degradation, leading to the attenuation of intestinal inflammation and cell apoptosis. Furthermore, to further ascertain the relationship of the signaling in regulating the biological behaviors of intestine under FK886 treatment, XMU-MP-1, the specific inhibitor of Hippo signaling, was applied. The reduced activity of NF-κB upon FK886 treatment was rescued by blocking Hippo signaling. Inhibition of Hippo signaling weakened the protective role of FK886 in sepsis-induced intestinal injury. Therefore, the inhibition of Visfatin may alleviate intestinal injury induced by sepsis via preventing inflammatory response and cell apoptosis through Hippo/YAP signaling pathway.

However, there are still some limitations in the present study. Despite the great importance of Hippo signaling underlying the protective role of FK886 in sepsis-induced intestinal injury was demonstrated in this research, the molecular mechanism concerning on how FK886 interacted with and regulated the Hippo signaling still remains unclear, which is deserved to be investigated in our further work. In addition, the exact upstream or downstream relationship between Hippo signaling and NF-κB signaling upon FK866 treatment in sepsis-mediated intestinal injury was not concentrated on in this study, and the subsequent research in this aspect is also urgently required in our future work.

## Conclusions

In summary, these results indicate that Visfatin plays an important role in sepsis and may serve as the target of sepsis treatment. Inhibition of Visfatin can alleviate inflammatory response and intestinal cell apoptosis in sepsis-induced intestinal injury, and regulate Hippo/NF-κB signaling pathway. This study not only enhances the understanding of the pathogenesis of sepsis-induced intestinal injury, but also provides new ideas for the treatment of sepsis.

## Data Availability

All data generated or analyzed during this study are included in this published article.
